# Measurement invariance and differential item functioning of the positive and negative affect schedule: a psychometric study in Ecuadorian young adults

**DOI:** 10.3389/fpsyg.2025.1635726

**Published:** 2025-09-12

**Authors:** Cesar Parra-Gaete, Andrea Vinueza-Cabezas, Mikaela Bourgeat-Salazar

**Affiliations:** ^1^CEC Research Group, Escuela de Psicología y Educación, Universidad de Las Américas, Quito, Ecuador; ^2^Grupo de Investigación Bienestar, Salud y Sociedad, Escuela de Psicología y Educación, Universidad de Las Américas, Quito, Ecuador

**Keywords:** positive and negative affect schedule (PANAS), item response theory (IRT), CFA, positive psychology, Ecuador, differential item functioning (DIF)

## Abstract

This study aimed to validate the two-factor structure of the Spanish version of the Positive and Negative Affect Schedule (PANAS) in a sample of Ecuadorian young adults, examining its reliability, construct validity, and measurement invariance across gender. A total of nine hundred and eighteen participants completed the PANAS, along with measures of personality traits and negative life events. Confirmatory factor analysis (CFA), measurement invariance testing, and Differential Item Functioning (DIF) analyses were conducted. The two-factor model showed excellent fit after removing the item Alerta (“Alert”), which exhibited poor loading likely due to contextual reinterpretation. Both Positive Affect (PA) and Negative Affect (NA) scales demonstrated strong internal consistency (α >0.89). Discriminant validity was supported by near-zero latent correlations and compliance with the Fornell–Larcker criterion. Partial metric and scalar invariance across gender were observed, with DIF analyses revealing item-level differences, especially for fear— and hostility—related emotions. Criterion validity was confirmed via expected correlations with life events and personality traits. The PANAS shows robust psychometric properties in this population, although some items exhibit gender-based variability in interpretation. Cultural sensitivity and periodic item review are essential in emotional assessment tools to ensure conceptual and contextual validity.

## Introduction

Mental health concerns have become increasingly prevalent across the global population, with young adults emerging as a particularly vulnerable group (Westberg et al., 2022; Zhang and Carciofo, 2021). These challenges are influenced by various factors, including the impact of social media (Wacks and Weinstein, 2021), the lingering psychological effects of the COVID-19 pandemic (Graupensperger et al., 2022), social isolation (Mann et al., 2022), academic and occupational stress (Ganson et al., 2021; Sivertsen et al., 2024), among others. As young adults navigate this critical and often turbulent stage of life, they encounter multiple stressors that contribute to emotional strain. Despite the growing need for support, relatively few individuals in this demographic seek or receive professional mental health services (Mei et al., 2020).

This underscores the importance of equipping young people with tools to manage their affect—or emotional state—and respond to emotional imbalances effectively. To do so, it is essential to rely on valid and reliable instruments that can accurately assess emotional experiences. One of the most widely used tools for this purpose is the Positive and Negative Affect Schedule (PANAS; Watson et al., 1988). Although originally developed in the United States, the PANAS has been adapted and validated in numerous cultural contexts, including several Latin American countries, highlighting its utility for both research and applied settings (García et al., 2019; Moreta-Herrera et al., 2021). This study focuses on evaluating the psychometric properties of the PANAS in Ecuadorian young people, including its validity across gender groups.

Affect refers to the basic experience of emotional phenomena, encompassing pleasant or unpleasant moods, which can be independent or attributed to a specific cause, initiating an emotional episode (Barrett and Bliss-Moreau, 2009; Rosenberg, 1998; Watson et al., 1999). Watson et al. (1988) developed the PANAS to measure two primary dimensions of this construct: Positive Affect (PA) and Negative Affect (NA). The scale consists of twenty items (10 for each dimension) rated on a 5-point Likert scale, with a two-factor structure that has been validated across diverse populations, including clinical (Díaz-García et al., 2020; Hovmand et al., 2023) and non-clinical samples (Dahiya and Rangnekar, 2019), as well as different age groups and cultures (Rush and Hofer, 2014; Watson et al., 1988).

The PANAS has broad applications in psychological research. In positive psychology, it assesses links between affect and well-being; in clinical psychology, it helps evaluate mood disorders such as depression and anxiety (Díaz-García et al., 2020); and in developmental psychology, it has been adapted for use with children and adolescents (Padros-Blazquez et al., 2023). It has also been used in cross-cultural research (Pires et al., 2013; Terracciano et al., 2006), though some studies note challenges in achieving full measurement invariance across cultures (Davis et al., 2022), underscoring the importance of cultural adaptations.

A substantial body of research has examined the connections between the Big Five personality traits (neuroticism, extraversion, conscientiousness, agreeableness, and openness) and the PANAS scale (Busseri and Erb, 2024; Díaz-García et al., 2020; Fagley, 2018; Vera-Villarroel et al., 2019). The most robust relationships emerge between neuroticism and NA, and between extraversion and PA. Studies consistently demonstrate that individuals scoring high in neuroticism report significantly greater NA, experiencing more frequent negative emotional states (Busseri and Erb, 2024; Díaz-García et al., 2020; Vera-Villarroel et al., 2019). Conversely, extraversion shows strong positive associations with PA, indicating that more extraverted individuals tend to experience heightened positive emotions (Busseri and Erb, 2024; Díaz-García et al., 2020; Vera-Villarroel et al., 2019). These associations reveal how personality shapes emotional experience and reinforce the PANAS's relevance in personality research.

Beyond personality, affect is influenced by life experiences. Research indicates that individuals who face more negative life events tend to report higher NA and show physiological indicators of stress, such as increased heart rate and decreased heart rate variability (Parra-Gaete and Hermosa-Bosano, 2023; Schneider et al., 2021). These effects are evident both in immediate emotional responses and in longer-term patterns, as supported by longitudinal evidence (Cooke et al., 2022).

The relationship between affect and gender reveals complex and sometimes contradictory findings. For instance, research on schizophrenia spectrum disorders found that men exhibited slightly higher NA scores than women, suggesting gender-specific emotional responses in clinical samples (Mohn et al., 2018). Conversely, another study reported that men scored higher than women in PA on adolescents and young adults, indicating potential gender differences in the experience of positive affects (Ortuño-Sierra et al., 2015). However, a study of Spanish children found no significant gender differences in latent means for PA or NA, demonstrating that the PANAS measures affect consistently across genders in certain populations (Sanmartín et al., 2018).

Further complicating the interpretation of these findings, research on Differential Item Functioning (DIF) has shown that specific PANAS items may be interpreted differently by men and women, potentially influencing observed gender differences (Fung and Jin, 2023). Moreover, cultural context appears to moderate these patterns. For example, a study of Mexican adults found equivalent levels of PA and NA between genders, suggesting that cultural norms may mitigate or even eliminate gender differences in affective experiences (Moral de la Rubia, 2019).

Despite these variations, the PANAS has proven particularly valuable in this research due to its strong psychometric properties across diverse populations, reliably capturing stress-related emotional changes in both clinical and non-clinical samples (Crawford and Henry, 2004; Díaz-García et al., 2020). Recent validations using confirmatory factor analysis (CFA) reaffirm its two-factor structure in Indian youth (Kumar et al., 2025), and comparable results were reported when adapting the scale for Denmark patients with emotional health conditions (Hovmand et al., 2023). Nevertheless, discrepancies remain. In a large-scale study comparing Singaporean and American adults demonstrated that certain items were interpreted differently, leading to reduced reliability in the measurement (Lee et al., 2020). Likewise, a study in Korean college students proposed that two dimensions of NA resulted in a three-factor appropriate fit, the invariability in the results may be caused by cultural Korean ideologies (Park et al., 2022). Globally, the key outcomes from the studies reveal that this scale can only be effectively used if it has cultural adjustments. These findings highlight the importance of adapting the scale to specific cultural contexts.

In Ecuador, two prior validations of the PANAS supported its original two-factor model (García et al., 2019; Moreta-Herrera et al., 2021). A third study conducted by Sanmartín et al. (2020) explicitly tested measurement invariance across gender in an adolescent Ecuadorian sample and confirmed configural, metric, and scalar invariance. However, this study was limited to an adolescent population and did not include young adults (18 + age). Despite the scale's demonstrated robustness, important gaps remain regarding its cross-cultural applicability and the potential for gender-based measurement bias.

To address these gaps, the present study has three main objectives: (1) to validate the PANAS in a sample of young adults from Ecuador, (2) to evaluate measurement invariance across gender groups, and, if invariance is not supported, (3) to identify DIF to determine whether observed gender differences in affect reflect genuine emotional variation or are influenced by measurement bias.

In line with these objectives, we propose the following hypotheses:

To support the validation of the PANAS in Ecuadorian young adults, we propose the following hypotheses. Cross-cultural studies have consistently supported its two-factor structure (Crawford and Henry, 2004; Haywood et al., 2024; Kumar et al., 2025; Terracciano et al., 2003; Yildiz, 2024) and Latin-American research has confirmed its reliability and validity (Moriondo et al., 2012; Pires et al., 2013; Robles and Páez, 2003). In Ecuador, however, studies have primarily focused on adolescents (Sanmartín et al., 2020), university students (Moreta-Herrera et al., 2021), and broad community samples (García and Arias, 2019), leaving its factorial structure in young adults underexplored. This study addresses that gap.

**H1**. The original two-factor structure of the PANAS (PA and NA) is expected to show an adequate fit in Ecuadorian young adults.

Regarding gender, previous findings have shown higher NA scores in women, but no consistent differences in PA (Mohn et al., 2018; Ortuño-Sierra et al., 2015). Nonetheless, item-level analyses suggest possible gender-related bias (Medvedev et al., 2023), highlighting the need for invariance testing.

**H2**. The PANAS demonstrates invariance across gender among Ecuadorian young adults.

**H3**. In the absence of full invariance, it is expected that specific items will show DIF by gender.

## Method

### Sample

The study included a total of nine hundred and eighteen participants, recruited through a non-probabilistic convenience sampling via social media posts shared by community organizations and local networks, as well as word-of-mouth referrals. The gender distribution comprised 39.3% men (*n* = 361) and 60.7% women (*n* = 557), reflecting a higher representation of women in the sample. In terms of educational attainment, most participants were currently enrolled in undergraduate programs (64.6%, *n* = 593), followed by those who had completed a bachelor's degree (19.1%, *n* = 175). A smaller proportion had completed high school (11.4%, *n* = 105), a master's or doctoral degree (4.7%, *n* = 43), and only primary education (0.2%, *n* = 2).

Monthly income levels were relatively evenly distributed. The largest group reported earnings between USD 401 and 800 (25.9%, *n* = 238), followed closely by those earning USD 801 to 1500 (25.4%, *n* = 233). Others reported earnings of USD 1501 to 2000 (16.8%, *n* = 154), over USD 2000 (18.7%, *n* = 172), or between USD 81 and 400 (13.2%, *n* = 121). Regarding relationship status, 52.7% (*n* = 484) reported being single, while 47.3% (*n* = 434) were in a romantic relationship. This distribution suggests a balanced representation of individuals across different statuses. Participants' mean age was 23.31 years (*SD* = 5.4), indicating a predominantly young adult sample. Overall, the sample is characterized by a higher proportion of women, a majority of individuals currently pursuing tertiary education, and a diverse range of income levels and relationship statuses.

Given that gender is one of the most important variables in this study, sample analyses were conducted to determine whether sociodemographic variables were equally distributed across gender groups. For the age variable, no statistically significant difference (*t* = 1.42, *p* =.157) was found between men (mean = 23.62) and women (mean = 23.11). Regarding education level, a statistically significant difference with a small effect size was observed (χ^2^ = 19.64, gl = 4, *p* < 0.001, Cramér's V = 0.15), with more men represented in the highest education category and more women in undergraduate programs. For monthly income, a small but statistically significant difference was found (χ^2^ = 14.70, gl = 4, *p* = 0.005, Cramér's V = 0.13), with men more frequently represented in the highest income bracket. In the case of relationship status, a statistically significant difference with a small effect size was also observed (χ^2^ = 4.79, gl = 4, *p* = 0.028, Cramér's V = 0.07), with a higher proportion of women reporting being in a romantic relationship. It is important to note that although some of these differences reached statistical significance, all effect sizes were small, indicating a minor impact on the overall analysis. Complete analysis can be found in Table 1.

**Table 1:** Sociodemographic analysis by gender.

**Variable**	**Men**	**Women**	**χ^2^ (*p* value)**	**Cramér's V**
**Educational attainment**				
Master's or doctoral degree	27 (7.5%)	16 (2.9%)	19.63 (*p* < 0.001)	0.15
Bachelor's degree	82 (22.7%)	93 (16.7%)		
Undergraduate programs	209 (57.9%)	384 (68.9%)		
High school	43 (11.9%)	62 (11.1%)		
Primary education	0 (0%)	2 (0.4%)		
**Monthly income**
over USD 2000	87 (24.1%)	85 (15.3%)	14.7 (*p* = 0.005)	0.13
USD 1501 to 2000	52 (14.4%)	102 (18.3%)		
USD 801 to 1500	97 (26.9%)	136 (24.4%)		
USD 401 and 800	83 (23.0%)	155 (27.8%)		
USD 81 and 400	42 (11.6%)	79 (14.2%)		
**Relationship status**
Romantic relationship	154 (42.7%)	280 (50.3%)	4.79 (*p* = 0.028)	0.07
Single	207 (57.3%)	277 (49.7%)		

## Instruments

### Demographic questionnaire

The online survey was structured into three sections. The first section presented a brief introduction to the study, informed consent, and researcher contact information. The second section included screening questions regarding participants' age, spoken language, and place of residence; if participants did not meet the inclusion criteria, the questionnaire terminated. The final section assessed demographic variables such as gender, level of education, and monthly household income through multiple-choice items. Participants who met the eligibility criteria then proceeded to complete the PANAS scale, Negative Life Events Scale and Big Five test.

### Positive and Negative Affect Scale (PANAS)

The PANAS, developed by Watson et al. (1988), assesses an individual's inclination to approach life positively. In this study, this scale was used as a complementary measure. It consists of two sub-factors, each with ten items. The first sub-factor measures PA, while the second measures NA. Each item utilizes a five-point Likert scale (0 = not at all, 4 = very much), and a total score for each subscale is obtained by summing the responses for each item. This study used the Spanish adaptation by Sandín et al. 91999), which has reported internal consistency values of α = 0.89 for the PA subscale and α = 0.91 for the NA subscale. All items were presented in Spanish; English translations are provided in tables for interpretative purposes only.

### Student life events scale (ESVE-R)

The Spanish version (Martínez Correa and Reyes del Paso, 2003) of the questionnaire created by Linden (1984) was used to measure stressful events in the lives of university students over the past two years. This version includes forty four items that measure different types of stressful events (death of a close family member, accident or serious illness, drug or alcohol abuse, being incarcerated, etc.). Participants were required to indicate (1) the number of life events they had experienced over the past year from the list, ranging from 0 (Never) to 10 or more events, and (2) their rating of the perceived stressfulness of each event on a scale ranging from 0 (Not stressful at all) to 10 (Very stressful). The scale has reported test-retest reliability of .77 (Martínez Correa and Reyes del Paso, 2003).

### Big Five Scale Short Short Version (BFPTSQ)

The Big Five Personality Trait Short Questionnaire (BFPTSQ), a concise adaptation of the Big Five personality model developed by (Morizot, 2014), comprises fifty Likert-type items with five response options. This instrument assesses five key dimensions of personality, each demonstrating adequate internal consistency: agreeableness (α = 0.75), extraversion (α = 0.87), openness (α = 0.83), conscientiousness (α = 0.82), and emotional stability (α = 0.85; Ortet et al., 2017). The shortened version is preferred over the full test to minimize potential fatigue effects. For the purposes of this study, the Spanish adaptation of the questionnaire, developed by Ortet et al. (2017), has been utilized.

## Procedure

Participants were recruited through convenience sampling using social media posts shared by community organizations and local networks, as well as through word-of-mouth referrals. While some recruitment messages were distributed by educational platforms, participation was open to any young adult meeting the inclusion criteria. All participants were presented with an informed consent form, indicating that their participation was entirely voluntary and that they could terminate the questionnaire if they felt uncomfortable without any adverse consequences. Data collection occurred between October and December 2023.

The study was conducted following the Declaration of Helsinki and approved by the Ethics Committee of Pontificia Universidad Católica del Ecuador (EO-115-2022).

## Data analysis

To examine the construct validity of the PANAS, we performed Confirmatory Factor Analysis (CFA) using the lavaan package (Rosseel, 2012) in R (R Core Team, 2023). The sample was divided into two subsamples: a calibration sample (*n* = 367) for initial model testing and a validation sample (n = 551) for confirming subsequent model adjustments. Given the ordinal nature of the data and absence of multivariate normality, we employed Diagonally Weighted Least Squares (DWLS) estimation with polychoric correlation matrices.

Model fit was evaluated using established criteria (Hu and Bentler, 1999): comparative fit index (CFI ≥ 0.90), root mean square error of approximation (RMSEA ≤ 0.08 with 90% CI between 0.06 and 0.08), and standardized root mean square residual (SRMR ≤ 0.08). Reliability was assessed using Cronbach's alpha (Cronbach, 1951) and McDonald's omega (McDonald, 2013), with 0.70 as the minimum acceptable threshold.

For discriminant validity, we applied the Fornell-Larcker criterion by comparing the square root of the Average Variance Extracted (AVE) for each factor with the inter-factor correlations. Measurement invariance across gender groups was tested using multi-group CFA with the WLSMV estimator. When full invariance was not achieved, we examined partial invariance models and conducted DIF analysis using the mirt package (Phil et al., 2014) to identify potential gender-based response differences. In the case DIF was detected, we would develop a Shiny application to compute scores based on gender-specific item parameters. This app, built using the *shiny* package (Chang et al., 2025), is freely available on the Shinyapps.io platform.

To establish criterion validity, we examined correlations between PANAS factors and negative life events, hypothesizing positive associations for NA and inverse relationships for PA. Additionally, we analyzed relationships between PANAS scores and Big Five personality traits, anticipating NA to correlate inversely with emotional stability and conscientiousness, while PA would show positive associations with extraversion and openness. Figures to better explain the relationship between variables where done using the ggplot package (Wickham, 2016).

## Results

### Model fit

For the validation model fit indices, the item *Alerta* “Alert” was eliminated due to high cross-loadings on both factors. This may be attributed to the item having different connotations in the Ecuadorian context. After removing this item, the model suggested that the two-factor structure fits the data well. The CFI value was 0.974, and the TLI value was 0.969; both indexes are higher than the 0.95 threshold, indicating excellent fit. The RMSEA value was 0.060 (Lower *CI* = 0.054; Upper *CI* = 0.066), which falls within the acceptable range of less than 0.08. The SRMR was 0.059, below the 0.08 threshold, indicating good fit. Overall, these fit indices suggest that the model provides a good representation of the data. For the test model fit, the robust fit indices CFI = 0.973 and TLI = 0.968 suggest that the model performs well even when accounting for potential deviations from normality. The robust RMSEA = 0.063 (Lower CI = 0.057; Upper CI = 0.069) and SRMR = 0.06 also indicate good model fit. Descriptive statistics for all items, including means, standard deviations, minimum and maximum values, skewness, and kurtosis, are provided in Supplementary Table 1.

The reliability and validity of the PANAS scales were assessed using the ordinal version of the Cronbach's alpha, McDonald's omega, and AVE. For the NA scale, the Cronbach's alpha is 0.899, while McDonald's omega value was 0.863. The AVE for NA was 0.474, which is slightly below the ideal threshold of 0.50, but still acceptable given the high reliability coefficients. For the PA scale, Cronbach's alpha values was 0.924, and McDonald's omega was 0.912. The AVE for PA is 0.59, which exceeds the 0.50 threshold, indicating good convergent validity. Both scales demonstrate strong reliability, with ordinal alpha and omega values above 0.85, indicating excellent internal consistency.

All factor loadings for both NA and PA were statistically significant (*p* < 0.001), indicating that each item contributes meaningfully to its respective factor. The standardized factor loadings (Std. load) ranged from 0.552 (*Disgustado/a* “Disgusted”) to 0.812 (*Atemorizado/a* “Afraid”) for NA and from 0.514 (*Interesado/a* “Interested”) to 0.867 (*Inspirado/a* “Inspired”) for PA. These loadings suggest that the items are strong indicators of their respective latent factors. The high factor loadings provide evidence for the construct validity of the PANAS scales. The complete factor loadings can be found in Table 2.

**Table 2:** CFA loading.

	**Estimate**	**SE**	**Z**	***P*-value**	**Std.load**
**Negative affect**					
*Asustado/a* (Scared)	1.000	0.784	0.784		
*Atemorizado/a* (Afraid)	1.035	0.035	29.969	0.000	0.812
*Avergonzado/a* (Ashamed)	0.931	0.038	24.443	0.000	0.730
*Culpable* (Guilty)	0.793	0.042	19.007	0.000	0.622
*Disgustado/a* (Disgusted)	0.704	0.045	15.762	0.000	0.552
*Hostil* (Hostile)	0.713	0.047	15.190	0.000	0.559
*Irritable* (Irritable)	0.752	0.041	18.144	0.000	0.590
*Miedoso/a* (Fearful)	1.013	0.034	29.877	0.000	0.794
*Nervioso/a* (Nervous)	0.990	0.033	29.792	0.000	0.776
*Tenso/a* (Tense)	0.760	0.043	17.834	0.000	0.596
**Positive affect**					
*Activo/a* (Active)	1.000	0.832	0.832		
*Estimulado/a* (Excited)	0.751	0.032	23.152	0.000	0.625
*Motivado/a* (Motivated)	1.019	0.021	48.231	0.000	0.847
*Entusiasmado/a* (Enthusiastic)	1.001	0.021	47.789	0.000	0.832
*Orgulloso/a* (Proud)	0.816	0.028	29.415	0.000	0.679
*Inspirado/a* (Inspired)	1.042	0.020	51.074	0.000	0.867
*Decidido/a* (Determined)	1.008	0.022	46.499	0.000	0.839
*Atento/a* (Attentive)	0.960	0.025	38.703	0.000	0.799
*Interesado/a* (Interested)	0.618	0.037	16.819	0.000	0.514

Items were presented to participants in Spanish. English translations are provided in parentheses for interpretative purposes only.

The covariance between NA and PA was small and non-significant (*r* = −0.039, *p* = 0.403), indicating that the two factors are largely independent. This supports the theoretical distinction between PA and NA. Several residual correlations were significant, such as between *Atemorizado/a* (“Afraid”) and *Miedoso/a* (“Fearful”) (*r* = 0.474) and between *Disgustado/a* (“Disgusted”) and *Irritable* (“Irritable”) (*r* = 0.339). These residual correlations suggest that some items share additional variance beyond what is captured by the latent factors, possibly due to similar wording or content. While these correlations do not undermine the overall model fit, they highlight areas where the items may overlap in meaning.

The variances of the latent factors were significant (*p* < 0.001), indicating that the factors explain a substantial portion of the variance in the items. The residual variances of the items were also significant, indicating that some variance remains unexplained by the latent factors. This is expected in CFA models, as not all item variance is accounted for by the latent constructs.

The CFA results support the two-factor structure of the PANAS, with strong evidence for reliability and validity. The NA and PA scales demonstrate excellent internal consistency, and the factor loadings confirm that the items are strong indicators of their respective constructs.

### Discriminant validity

The Fornell-Larcker Criterion was used to further evaluate discriminant validity by comparing the square root of the AVE for each factor with the correlation between the factors. For discriminant validity to be established, the square root of the AVE for each factor must be greater than the correlation between the factors. The square root of the AVE for NA was 0.689, and for PA, it is 0.768. The correlation between NA and PA was −0.039, which is very small and non-significant. Because both 0.689 and 0.768 exceed −0.039, the Fornell-Larcker Criterion is satisfied, supporting the discriminant validity of both PANAS subscales.

### Measurement invariance

The configural model, which tests whether the basic factor structure is equivalent across gender groups, demonstrated acceptable fit (CFI = 0.973, TLI = 0.968, RMSEA = 0.064, SRMR = 0.068). These results indicate that both men and women conceptualize PA and NA similarly, with the same items loading on their respective factors across groups (see Table 3).

**Table 3:** Model comparison.

	**Chisq**	**Chisq**	**diff**	**Df**	***p*-value**	**CFI**	**TLI**	**RMSEA**	**SRMR**
Configural	292	555.93				0.973	0.968	0.064	0.068
Metric	306	597.56	23.576	14	0.05151	0.973	0.97	0.062	0.069
Scalar	358	642.11	68.299	52	0.06422	0.971	0.972	0.06	0.068
Structural	360	652.27	1.236	2	0.53916	0.974	0.975	0.056	0.068

When testing for metric invariance (equality of factor loadings), the fully constrained model showed a significant deterioration in fit (Δχ^2^ = 39.63, df = 17, *p* = 0.001). Closer examination revealed three items from the NA scale with non-invariant loadings: *Irritable* “Irritable” (difference = 0.16), *Hostil* “Hostile” (difference = 0.12), and *Culpable* “Guilty” (difference = 0.12). This suggests that these particular items may be interpreted or responded to differently by men and women. After establishing partial metric invariance by freeing these three loadings, model fit remained acceptable (CFI = 0.973, TLI = 0.970, RMSEA = 0.062, SRMR = 0.069) with no significant deterioration compared to the configural model (χ^2^ = 23.58, df = 14, *p* = 0.05). Testing for scalar invariance (equality of item thresholds) revealed additional non-invariance (Chi = 658.98, Chi dif = 91.09, df = 55, *p* = 0.001), particularly for *Miedoso/a* “Fearful” (threshold differences = 0.47 and 0.38) and *Estimulado* “Excited” (threshold difference = 0.37). After establishing partial scalar invariance by freeing these thresholds, model fit remained adequate (CFI = 0.971, TLI = 0.972, RMSEA = 0.060, SRMR = 0.068) with no significant deterioration from the metric model (χ^2^ = 68.30, df = 52, *p* = 0.06; see Table 3).

The final test of structural invariance (equality of factor variances and covariances) showed excellent model fit (CFI = 0.974, TLI = 0.975, RMSEA = 0.056, SRMR = 0.068) with no significant deterioration from the scalar model (χ^2^ = 1.24, df = 2, p = 0.54; see Table 3), indicating that the relationships between the latent factors are equivalent across gender groups. The establishment of partial metric and scalar invariance suggests that while most items function similarly across genders, some notable exceptions exist. The non-invariant items (*Irritable* “Irritable”, *Hostil* “Hostiles”, *Culpable* “Guilty”, *Miedoso/a* “Fearful”, and *Estimulado/a* “Excited”) may reflect genuine gender differences in emotional expression or interpretation.

### Differential item functioning analysis

For the NA scale, several items showed statistically significant DIF (*p* < 0.05), including *Asustado/a* “Scared” (χ^2^ = 11.28, *p* = 0.046), *Atemorizado/a* “Afraid” (χ^2^ = 13.21, *p* = 0.021), *Hostil* “Hostile” (χ^2^ = 15.49, *p* = 0.008), *Irritable* “Irritable” (χ^2^ = 11.33, *p* = 0.045), and Miedoso/a “Fearful” *(*χ^2^ = 16.27, *p* = 0.006). These items demonstrated notable differences in both discrimination (a) parameters and threshold (b) parameters between men and women. For instance, Miedoso/a “Fearful” showed higher discrimination in women (*a* = 3.24) compared to men (*a* = 2.49), suggesting this item better differentiates between individuals with varying levels of NA in women (see Table 4). Similarly, threshold parameters for *Hostil* “Hostile” were substantially higher for men across all response categories, indicating men require greater levels of latent NA to endorse higher response options for this item.

**Table 4:** DIF analysis results.

**Model**	**AIC**	**SABIC**	**BIC**	**X2**	***p***	**Men**					**Women**
						**a**	***b1***	***b2***	***b3***	***b4***	***a***	***b1***	**b2**	**b3**	**b4**
**Negative affect**															
*Asustado/a* (Scared)	−1.275	4.411	20.284	11.275	0.046	1.990	−0.390	0.875	1.628	2.365	2.253	−0.703	0.463	1.318	2.362
*Atemorizado/a* (Afraid)	−3.209	2.477	18.349	13.209	0.021	2.431	−0.078	0.944	1.727	2.263	3.682	−0.325	0.613	1.273	2.001
*Avergonzado*/*a* (Ashamed)	4.886	10.572	26.444	5.114	0.402	2.144	−0.297	0.883	1.753	2.892	1.693	−0.224	0.971	1.834	2.777
*Culpable* (Guilty)	0.494	6.181	22.053	9.506	0.091	1.668	−0.181	0.970	1.602	2.701	1.102	0.022	1.745	2.650	3.863
*Disgustado/a* (Disgusted)	8.675	14.361	30.233	1.325	0.932	1.378	−1.172	0.955	1.916	3.400	1.133	−1.383	1.071	2.150	3.694
*Hostil* (Hostile)	−5.488	0.199	16.071	15.488	0.008	1.363	−0.494	1.091	2.475	3.369	0.897	−0.015	2.080	3.406	5.113
*Irritable* (Irritable)	−1.333	4.354	20.226	11.333	0.045	1.722	−0.779	0.689	1.523	2.659	1.052	−1.276	0.835	2.146	3.116
*Miedoso/a* (Fearful)	−6.265	−0.578	15.294	16.265	0.006	2.493	−0.402	0.970	1.678	2.366	3.243	−0.639	0.418	1.095	1.974
*Nervioso/a* (Nervous)	5.160	10.847	26.719	4.84	0.436	1.732	−1.243	0.140	1.071	2.230	2.340	−1.078	0.007	0.821	1.721
*Tenso/a* (Tense)	1.052	6.739	22.611	8.948	0.111	1.611	−1.936	0.147	1.200	2.523	1.137	−2.486	−0.071	1.499	2.670
**Positive affect**															
*Activo/a* (Active)	5.515	11.202	27.074	4.485	0.482	2.413	−2.340	−1.108	0.032	1.203	2.840	−1.984	−0.865	0.209	1.116
*Estimulado/a* (Excited)	1.784	7.471	23.343	8.216	0.145	1.401	−2.604	−0.178	1.146	3.105	1.495	−1.899	−0.177	1.055	2.472
*Motivado/a* (Motivated)	4.971	10.657	26.529	5.029	0.412	2.589	−1.854	−0.827	0.263	1.502	3.006	−2.027	−0.756	0.252	1.262
*Entusiasmado*/a (Enthusiastic)	9.271	14.958	30.830	0.729	0.981	2.773	−2.056	−0.835	0.241	1.249	2.896	−1.998	−0.720	0.277	1.277
*Orgulloso/a* (Proud)	4.916	10.603	26.475	5.084	0.406	1.639	−1.912	−0.908	0.315	1.573	1.738	−1.950	−0.667	0.260	1.507
*Inspirado/a* (Inspired)	6.687	12.374	28.246	3.313	0.652	3.361	−1.745	−0.764	0.200	1.272	3.219	−1.559	−0.635	0.276	1.189
*Decidido/a* (Determined)	2.888	8.574	24.446	7.112	0.212	2.752	−2.115	−1.195	−0.022	1.082	2.669	−2.036	−0.848	0.189	1.179
*Atento/a* (Attentive)	0.704	6.390	22.263	9.296	0.098	2.012	−2.370	−1.104	0.036	1.455	2.872	−2.248	−1.021	0.030	1.043
*Interesado/a* (Interested)	−4.705	0.981	16.853	14.705	0.012	1.062	−2.050	−0.615	0.564	3.186	1.147	−2.105	−0.593	0.975	2.477

Chi squared has 5 degrees of freedom. Items were presented to participants in Spanish. English translations are provided in parentheses for interpretative purposes only.

In contrast, the PA scale showed greater measurement equivalence across genders, with only *Interesado/a* “Interested” exhibiting significant DIF (χ^2^ = 14.71, *p* = 0.012). This item displayed slightly higher discrimination in women (*a* = 1.15) compared to men (*a* = 1.06), along with systematically lower thresholds, suggesting women may be more likely to endorse higher response categories at equivalent levels of PA. The remaining PA items showed non-significant DIF (*p* > 0.05), supporting their measurement invariance across gender groups (see Table 4).

These findings complement and extend the results from the measurement invariance analysis, providing more granular evidence about specific items that function differently for men and women. The DIF results particularly highlight that several NA items related to fear (*Miedoso/a* “Fearful”, *Asustado/a* “Scared”, *Atemorizado/a* “Afraid”) and hostility (Hostil “Hostile”, *Irritable* “Irritable”) show gender-based differences in how they relate to the underlying latent trait. This suggests that these emotional states may be experienced, interpreted, or reported differently by men and women, possibly reflecting sociocultural norms around emotional expression. The relative stability of PA items (with the exception of *Interesado/a* “Interested”) indicates that positive emotional states are more consistently measured across genders in this instrument. Complete DIF analysis and coefficients results for both factors across genders can be found in Table 4.

### Criterion validity

**Figure 1:**
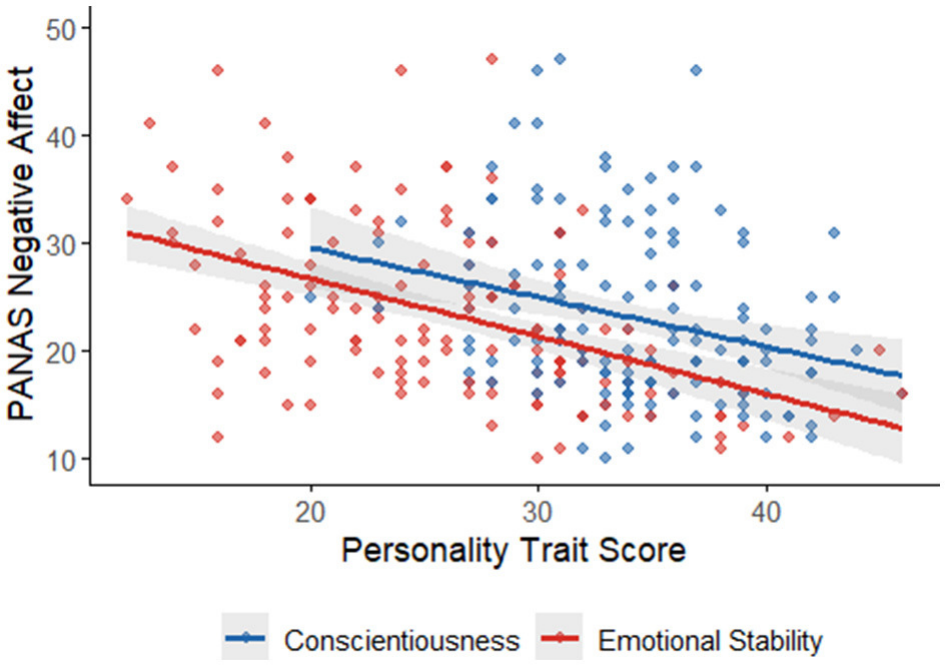
PANAS NA and the Negative Life Event Scores.

The correlation analysis revealed significant relationships between the PANAS scores and the negative life event factors. PANAS NA showed moderate positive correlations with both Frequency (*r* = 0.22, *p* < 0.001; See Figure 1) and Impact (*r* = 0.24, *p* < 0.001; See Figure 1), indicating that individuals with higher NA tend to report more frequent and impactful negative life events. In contrast, PANAS PA showed weak and non-significant correlations with both Frequency (*r* = 0.05, *p* = 0.16) and Impact (*r* = 0.07, *p* = 0.05), suggesting that PA is not strongly related to the frequency or impact of negative life events. Additionally, the strong positive correlation between Frequency and Impact (*r* = 0.67, *p* < 0.001) indicates that individuals who experience more frequent negative life events also tend to perceive them as more impactful. Overall, these results support the criterion validity of the PANAS NA scale, as it is meaningfully associated with external measures of negative life events, while the PANAS PA scale shows limited association in this context.

**Figure 2:**
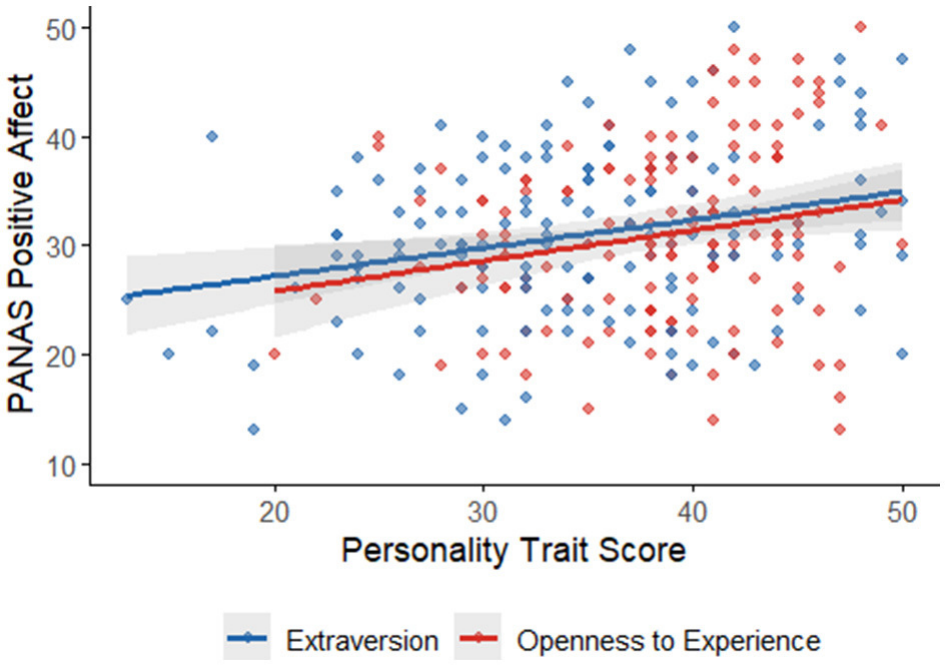
PANAS NA and BFP factors.

**Figure 3:**
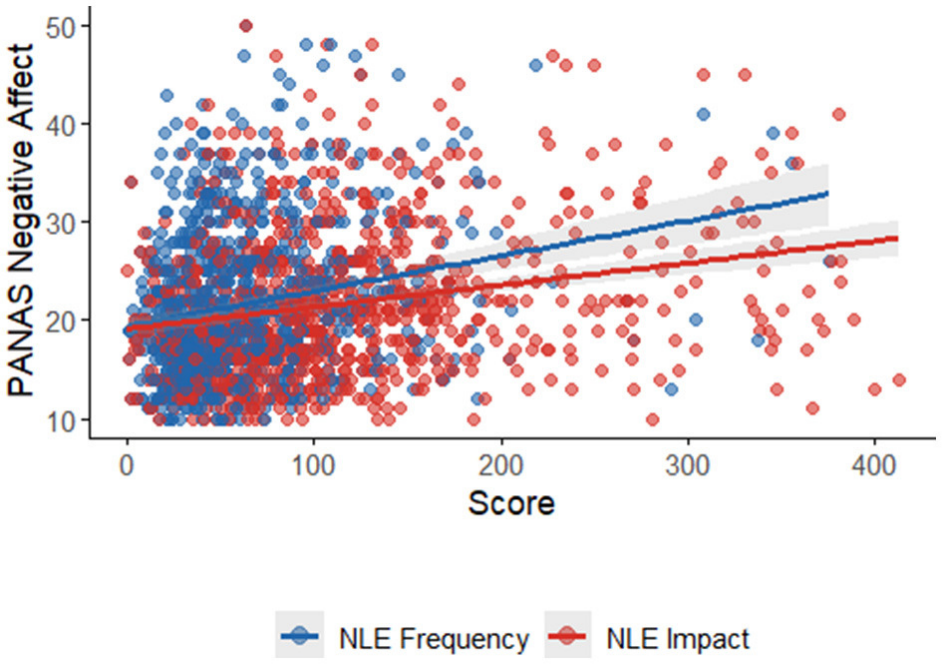
PANAS PA and BFP factors.

The correlation analysis revealed significant relationships between the PANAS scores and the Big Five personality traits. PANAS NA showed strong negative correlations with Emotional Stability (*r* = −0.49, *p* < 0.001; see Figure 2) and Conscientiousness (r = −0.28, *p* < 0.001; see Figure 2) indicating that individuals with higher NA tend to have lower emotional stability and lower conscientiousness. PANAS NA also showed weak negative correlations with Extraversion (*r* = −0.16, *p* = 0.06), and Agreeableness (*r* = −0.13, *p* = 0.13), though these were not statistically significant. In contrast, PANAS PA showed moderate positive correlations with Extraversion (*r* = 0.26, *p* < 0.001; see Figure 3) and Openness to Experience (*r* = 0.21, *p* = 0.01; see Figure 3), suggesting that individuals with higher PA tend to be more extraverted and open to new experiences. PANAS PA also showed weak positive correlations with Emotional Stability (*r* = 0.16, *p* = 0.049) and Conscientiousness (*r* = 0.13, *p* = 0.13), though the latter was not statistically significant. Overall, these results highlight meaningful relationships between affect and personality traits, with NA being strongly linked to lower emotional stability and conscientiousness, and PA being associated with higher extraversion and openness.

## APP development for test scoring

Based on the results of the DIF analysis, we developed a latent trait calculator using the graded response model and maximum likelihood estimation. The calculator employs gender-specific item parameters—estimated separately for men and women—to compute individual latent trait scores. These scores are then transformed into a more interpretable metric with a mean of 50 and a standard deviation of 10, facilitating understanding among non-technical users. To enhance interpretability, the application also includes a normal distribution graph that visually situates the individual score in relation to the population. Additionally, the standard error of measurement is computed for each estimated θ using the inverse square root of the Fisher information formula, providing an index of precision for each estimate.

## Discussion

The first hypothesis of this study proposed that the original two-factor structure of the PANAS (Positive Affect and Negative Affect) would show an adequate fit in Ecuadorian young adults. It was evaluated through analyses of construct validity, discriminant validity, criterion validity, and reliability. CFA provided strong evidence of construct validity, supporting the two-factor model with excellent fit indices after removing the item *Alerta* (“Alert”) due to its low factor loading, likely caused by semantic ambiguity in the Ecuadorian context. While previous validations of the PANAS in Ecuador retained this item (García et al., 2019; Moreta-Herrera et al., 2021), closer inspection reveals that it had the lowest loading (e.g., 31 in some cases). Similar concerns have been noted in other cultural settings, where *Alerta* (“Alert”) has demonstrated weak saturation on the Positive Affect factor—particularly among women—and has shown associations with symptoms of emotional distress rather than with positive activation (Dufey and Fernandez, 2012; López-Gómez et al., 2015; Vázquez and Hervás, 2010). These findings suggest that the adjective *Alerta* (“Alert”) may not consistently be interpreted as energizing or pleasant across all contexts. In Ecuador, this ambiguity may be amplified by recent sociocultural shifts, particularly the country's transition from being one of the safest to one of the most violent in Latin America, which may have altered the connotation of *Alerta* (“Alert”) toward heightened vigilance or fear. One proposed solution is to replace the term with *Despierto* (“awake”), which has demonstrated good psychometric properties (López-Gómez et al., 2015). However, without a thorough qualitative analysis within the target population, it is premature to conclude that this is the most appropriate alternative, especially when other terms such as *Atento* (“attentive”) or *Enérgico* (“energetic”) may better capture the intended meaning. These results highlight the importance of reassessing item functioning in consideration of evolving cultural and societal meanings.

Reliability estimates were uniformly high for both (α = 0.924, ω = 0.912) and NA (α = 0.899, ω = 0.863), in line with previous Ecuadorian studies and the original Spanish validation (Sandín et al., 1999). Although the AVE for NA (0.474) fell slightly below the recommended.50 cutoff, its strong internal consistency and substantial factor loadings (0.55–0.81) suggest that the construct is still acceptably measured. This result could indicate that NA, as a construct, encompasses more heterogeneous emotional expressions, which slightly reduces the shared variance among items. This interpretation aligns with findings by Rush and Hofer (2014), who observed that model fit significantly improved when specific item correlations, particularly among NA items, were allowed. Their results suggest that some NA items share variance not fully explained by the latent NA factor itself, likely due to their belonging to distinct mood content categories. This finding underscores the conceptual heterogeneity of NA and the potential for reduced cohesion among its indicators. In contrast, the AVE for PA (0.59) indicates good convergent validity, supporting the coherence of the PA items in capturing the intended construct. Discriminant validity was also supported by a near-zero latent correlation between PA and NA (*r* = – 0.04, p = 0.40) and by meeting the Fornell–Larcker criterion (√AVE_PA = 0.77, √AVE_NA = 0.69), reinforcing the theoretical independence of the two dimensions, as conceptualized initially by Watson et al. (1988).

Criterion validity was supported by moderate positive correlations between NA and both the frequency (*r* = 0.22, *p* < 0.001) and impact (*r* = 0.24, *p* < 0.001) of Negative Life Events (NLEs), suggesting that individuals with higher NA tend to report more frequent and impactful negative experiences. PA, by contrast, showed no significant associations with these variables, supporting the notion that NA is more reactive to stressors, while PA reflects a more stable, dispositional baseline less influenced by adversity (Crawford and Henry, 2004). Additionally, PA correlated positively with Extraversion (*r* = 0.26, *p* < 0.001) and Openness (*r* = 0.21, *p* = 0.01), while NA showed negative correlations with Emotional Stability (*r* = −0.49, *p* < 0.001) and Conscientiousness (*r* = −0.28, *p* < 0.001), replicating established affect–personality associations (Díaz-García et al., 2020; Fagley, 2018). These findings underscore the PANAS's utility not only as a descriptive tool but also as a predictor of affectively relevant psychological dispositions.

Regarding the second hypothesis that the PANAS demonstrates invariance across gender among Ecuadorian young adults this was partially supported. Measurement invariance testing revealed configural equivalence across gender, indicating that both men and women conceptualize PA and NA similarly. Partial metric and scalar invariance were established after freeing the loadings of *Hostil* “Hostile”, *Irritable* “Irritable”, and *Culpable* “Guilty”, as well as the thresholds for *Miedoso/a* “Fearful” and *Estimulado/a* “Excited”. This finding suggests that certain affective expressions may be interpreted or experienced differently by gender groups (Vandenberg and Lance, 2000). These non-invariant items, primarily associated with hostility and fear, may reflect sociocultural norms around emotional expressivity or interpretation in the Ecuadorian context. Nonetheless, the establishment of partial invariance supports valid latent mean comparisons across gender (Byrne et al., 1989). These results also highlight the importance of balancing statistical rigor with conceptual interpretability when determining invariance in culturally sensitive assessments.

Given that full invariance was not supported, we proceeded to test the third hypothesis: that specific items would exhibit Differential Item Functioning (DIF) by gender. DIF analysis provided further insight into gender-based item-level differences, revealing DIF in several NA items (*Asustado/a* “Scared”, *Atemorizado/a* “Afraid”, *Hostil* “Hostil”, *Irritable* “Irritable,” and *Miedoso/a* “Fearful, and one PA item *Interesado/a* “Interested”). Higher discrimination parameters for fear-related items among women suggest greater sensitivity of these items to variations in NA in female respondents. In contrast, threshold differences for *Hostil* “Hostil”, indicate that men require higher levels of underlying NA to endorse hostile feelings. These findings suggest that emotional states related to fear and hostility may be experienced or reported differently by men and women in Ecuador. This pattern may reflect traditional gender roles, which tend to socialize women to be more attuned to fear and anxiety making them more sensitive discriminators of NA while encouraging men to express aggression more readily (McLean and Anderson, 2009). However, because aggression is often more socially acceptable for men, they may require higher levels of NA to report the most intense expressions of hostility. Conversely, the relative consistency of PA items except for *Interesado/a* “Interested” suggests that positive emotions are measured more uniformly across gender. The exception may be explained by gender norms that expect women to more openly express emotional interest and engagement (Chaplin, 2015).

Several limitations of this study should be acknowledged. Although the sample offers valuable insights, it may not fully represent the broader Ecuadorian population due to its online, self-selected nature. Individuals from lower socioeconomic and educational backgrounds were underrepresented, particularly given that approximately half of Ecuadorian families earn less than $877 per month [Instituto Nacional de Estadística y Censos (INEC), 2025]. While anonymity likely reduced social desirability bias, its influence on responses cannot be entirely ruled out. Future research should explore the psychometric properties of the PANAS in more diverse samples, including varied age groups, educational levels, and geographic regions, while also controlling for social desirability bias. Replicating the study with older adults and clinical populations would further enhance generalizability. Additionally, the absence of qualitative methods such as interviews or focus groups is a notable limitation. These methods could offer deeper insights into participants' interpretations of specific items and clarify the observed gender-based DIF in certain Negative Affect indicators. For instance, they could help determine whether the item *Alerta* (“Alert”) hypothesized here to reflect fear or hypervigilance has indeed undergone a semantic shift in the Ecuadorian context. Such qualitative approaches would provide culturally grounded explanations for item functioning that cannot be captured through quantitative analysis alone.

These results highlight not only psychometric considerations but also the ethical imperative of adapting psychological instruments to evolving cultural realities. Ensuring semantic and contextual relevance is crucial to avoid perpetuating outdated or biased constructs, especially when used for clinical or educational decision-making. As a partial response to the findings presented in this article, we developed a Shiny app to help address the limitations identified, particularly the lack of full measurement invariance across genders. Detailed in the Results section, the app estimates a participant's latent trait using IRT parameters, providing more precise and individualized scores. It also offers a brief interpretation and a visual representation of how the score deviates from the population mean. By integrating these features, the app allows psychologists to continue using the PANAS in the Ecuadorian context while accounting for measurement bias. Although intended as a temporary solution, we hope it remains useful until a more culturally adapted version of the PANAS is developed through a comprehensive mixed-methods study. We invite you to explore the application at the following link: https://5lnpzo-cesar-parra.shinyapps.io/PANAS_APP/.

In conclusion, the PANAS demonstrates strong psychometric properties among Ecuadorian young adults, confirming its two-factor structure and high reliability following the removal of the culturally ambiguous item *Alerta* “Alert”. The observed partial measurement invariance and DIF across gender highlight the importance of cultural sensitivity and careful interpretation of specific items when assessing PA and NA in this population. These findings contribute to the growing literature on the cross-cultural validation of psychological instruments, underscoring the need to examine both universal and culture-specific dimensions of emotional assessment.

## Data Availability

The raw data supporting the conclusions of this article will be made available by the authors, without undue reservation.
