# Correction: Measurement invariance and differential item functioning of the positive and negative affect schedule: a psychometric study in Ecuadorian young adults

**DOI:** 10.3389/fpsyg.2025.1713425

**Published:** 2025-10-10

**Authors:** Cesar Parra-Gaete, Andrea Vinueza-Cabezas, Mikaela Bourgeat-Salazar

**Affiliations:** ^1^CEC Research Group, Escuela de Psicología y Educación, Universidad de Las Américas, Quito, Ecuador; ^2^Grupo de Investigación Bienestar, Salud y Sociedad, Escuela de Psicología y Educación, Universidad de Las Américas, Quito, Ecuador

**Keywords:** positive and negative affect schedule (PANAS), item response theory (IRT), CFA, positive psychology, Ecuador, differential item functioning (DIF)

Author Andrea Vinueza-Cabezas was erroneously spelled as Andrea Vinueza.

The original version of this article has been updated.

