# Supplementary material for: Measurement invariance and differential item functioning of the positive and negative affect schedule: a psychometric study in Ecuadorian young adults
Source: Front Psychol. 2025 Sep 12;16:1635726. doi: 10.3389/fpsyg.2025.1635726 (PMC12463610; doi:10.3389/fpsyg.2025.1635726)
Supplement: Supplementary file 1 [file Table_1.docx]

**Supplementary Material**

**Supplementary Table 1**

*PANAS Descriptive Statics*

| Item | Mean | SD | Min | Max | Skew | Kurtosis |
| --- | --- | --- | --- | --- | --- | --- |
| Negative Affect |  |  |  |  |  |  |
| *Asustado/a* (Scared) | 2.15 | 1.1 | 1 | 5 | 0.84 | -0.02 |
| *Atemorizado/a* (Afraid) | 1.97 | 1.09 | 1 | 5 | 1.1 | 0.52 |
| *Avergonzado/a* (Ashamed) | 1.92 | 1.03 | 1 | 5 | 1.09 | 0.62 |
| *Culpable* (Guilty) | 1.84 | 1.06 | 1 | 5 | 1.3 | 1.01 |
| *Disgustado/a* (Disgusted) | 2.21 | 0.97 | 1 | 5 | 0.86 | 0.4 |
| *Hostil* (Hostile) | 1.82 | 0.95 | 1 | 5 | 1.23 | 1.28 |
| *Irritable* (Irritable) | 2.24 | 1.08 | 1 | 5 | 0.84 | 0.15 |
| *Miedoso/a* (Fearful) | 2.14 | 1.09 | 1 | 5 | 0.9 | 0.15 |
| *Nervioso/a* (Nervous) | 2.62 | 1.18 | 1 | 5 | 0.38 | -0.75 |
| *Tenso/a* (Tense) | 2.67 | 1.03 | 1 | 5 | 0.57 | -0.23 |
| Positive Affect |  |  |  |  |  |  |
| *Activo/a (*Active*)* | 3.36 | 1.08 | 1 | 5 | -0.13 | -0.7 |
| *Estimulado/a (*Excited*)* | 2.72 | 1 | 1 | 5 | 0.3 | -0.49 |
| *Motivado/a (*Motivated*)* | 3.23 | 1.07 | 1 | 5 | -0.11 | -0.66 |
| *Entusiasmado/a (*Enthusiastic*)* | 3.24 | 1.08 | 1 | 5 | -0.02 | -0.75 |
| *Orgulloso/a (*Proud*)* | 3.15 | 1.17 | 1 | 5 | -0.13 | -0.84 |
| *Inspirado/a (*Inspired*)* | 3.2 | 1.15 | 1 | 5 | -0.13 | -0.79 |
| *Decidido/a (*Determined*)* | 3.38 | 1.08 | 1 | 5 | -0.18 | -0.69 |
| *Atento/a (*Attentive*)* | 3.42 | 1.05 | 1 | 5 | -0.2 | -0.65 |
| *Interesado/a (*Interested*)* | 2.85 | 1.14 | 1 | 5 | 0.03 | -0.86 |
| *Alerta (Alert)* | 2.9 | 1.13 | 1 | 5 | 0.16 | -0.74 |
